# Ten simple rules for an inclusive summer coding program for non-computer-science undergraduates

**DOI:** 10.1371/journal.pcbi.1007833

**Published:** 2020-09-03

**Authors:** Pleuni Pennings, Mayra M. Banuelos, Francisca L. Catalan, Victoria R. Caudill, Bozhidar Chakalov, Selena Hernandez, Jeanice Jones, Chinomnso Okorie, Sepideh Modrek, Rori Rohlfs, Nicole Adelstein

**Affiliations:** 1 San Francisco State University, San Francisco, California, United States of America; 2 Brown University, Providence, Rhode Island, United States of America; 3 University of California-San Francisco, San Francisco, California, United States of America; 4 University of Oregon, Eugene, Oregon, United States of America; 5 Aspire Vanguard College Preparatory Academy, Modesto, California, United States of America; Carnegie Mellon University, UNITED STATES

## Abstract

Since 2015, we have run a free 9-week summer program that provides non-computer science (CS) undergraduates at San Francisco State University (SFSU) with experience in coding and doing research. Undergraduate research experiences remain very limited at SFSU and elsewhere, so the summer program provides opportunities for many more students beyond the mentoring capacity of our university laboratories. In addition, we were concerned that many students from historically underrepresented (HU) groups may be unable to take advantage of traditional summer research programs because these programs require students to relocate or be available full time, which is not feasible for students who have family, work, or housing commitments. Our program, which is local and part-time, serves about 5 times as many students as a typical National Science Foundation (NSF) Research Experiences for Undergraduates (REU) program, on a smaller budget. Based on our experiences, we present 10 simple rules for busy faculty who want to create similar programs to engage non-CS HU undergraduates in computational research. Note that while some of the strategies we implement are based on evidence-based publications in the social sciences or education research literature, the original suggestions we make here are based on our trial-and-error experiences, rather than formal hypothesis testing.

## Structure of the program

The structure of the program is its most important characteristic. The first 6 rules are related to ways in which the structure of the program and the application process promote inclusivity and garner engagement.

### 1. Create a free part-time program with a defined schedule

Since many students at our campus (San Francisco State University [SFSU]) have a job or jobs, commute long distances, take care of family members, take summer classes, or volunteer in their community, our program is part-time (10 hours per week). The part-time nature allows students to participate while still tending to these other obligations. We decide on and set all meeting times at the time of application and have students apply to their desired meeting times. We make it clear that no work is expected outside of these hours. Once accepted to the program, students know their time obligation and schedule. For example, an applicant might be offered a spot on a team that meets from 10 am to 12 pm four days a week, in addition to an all-hands lunch meeting from 12 pm to 2 pm one day a week. So far, while the program is free to participants (thanks, in part to start-up funds and external grants), we have not been able to offer participants stipends. While we would prefer to offer students financial support, since we do not offer a federally funded stipend, we avoid any need to talk about citizenship. This makes our program more accessible to undocumented students compared with most federally funded summer research programs.

### 2. Remove barriers to application

Our application process starts early in spring and aims to attract a diverse cohort of students. We use a number of evidence-based recruitment strategies, including the following.

**Fig 1:**
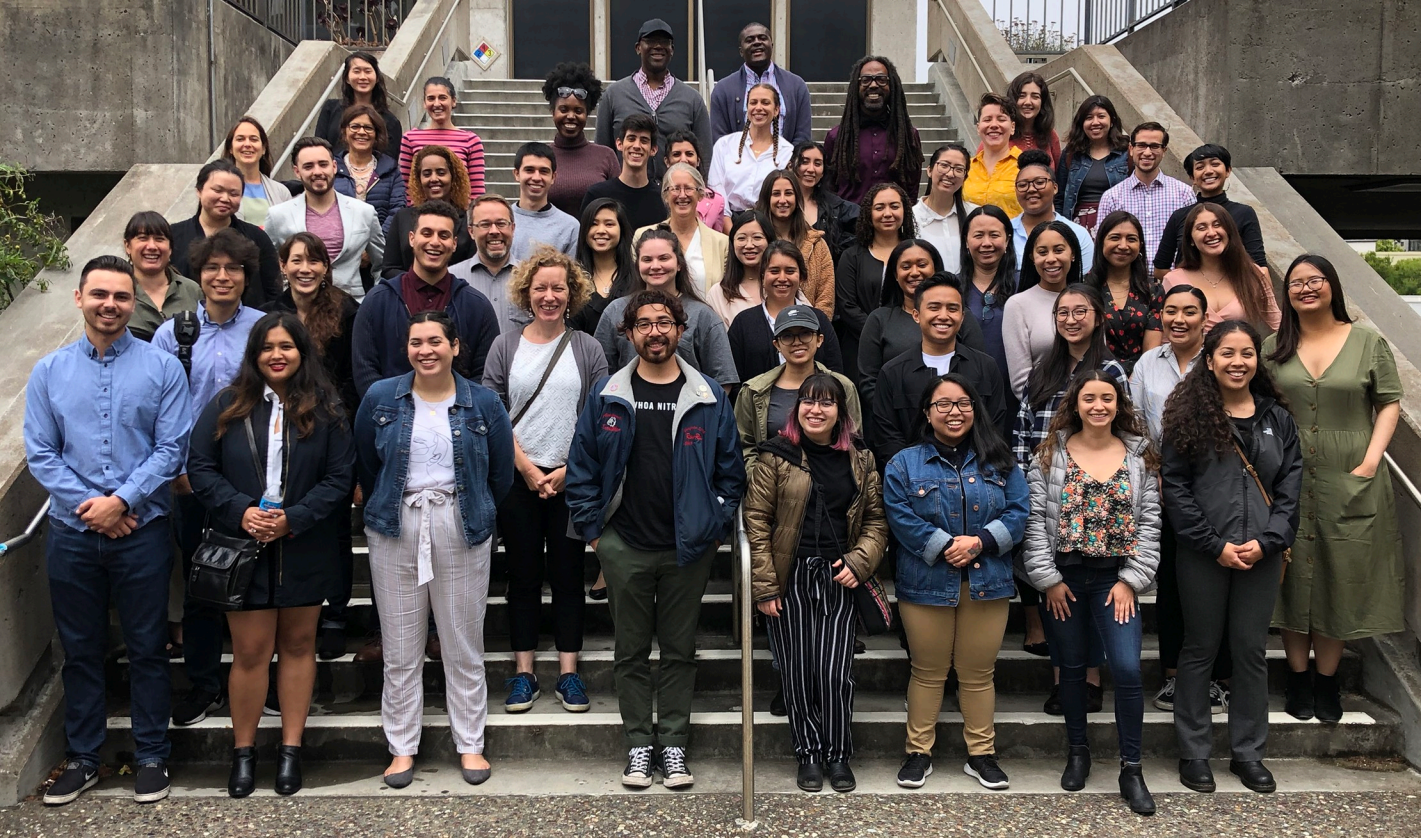
Group photo of the participants, mentors, and faculty of the 2019 Big Data Summer Program at the Summer Research Symposium. Photo by Dr. Blake Riggs, SFSU.

1. We emphasize that no prior knowledge is expected, there are no grade point average (GPA) or prerequisite requirements, and no letters of recommendation are needed.
2. We make it clear that the program is free.
3. On the flyer we use to advertise the program, we feature a visibly diverse group photo including virtually all of the previous year’s participants (similar to Fig 1).
4. We keep our online application very simple. The primary evaluation question is “What do you hope to gain from the summer program?”

In addition to passing out flyers, program faculty encourage individual students they know to apply. We evaluate applications based on student potential (including their interest in integrating computational research into their studies or career, and how much longer they will be at SFSU to continue to develop their skills under mentorship) and logistics (whether they are available for possible team meeting times). Perhaps in part because of these strategies, our applicant pool and selected student body have always consisted of large numbers of historically underrepresented (HU) groups (approximately 70% in 2018) and women and gender nonconforming (approximately 80% in 2018) students. These percentages are much higher than those for SFSU’s biology, chemistry, and economics departments, from which the program draws most of its applicants.

### 3. Create mentored teams of students

When working alone, we’ve often seen students learning computer science (CS) and programming get stuck on technical problems, leaving many feeling lost and inadequate and wanting to discontinue learning this new skill. Working in a mentored team, however, students have access to immediate support from their peers and mentor. This helps them learn technical skills more efficiently, develop relationships with each other, and cultivate a shared sense of belonging in computational research [1]. We recommend that each participant in a CS summer program be assigned to a team of 4 to 5 students with similar technical skill levels led by a near-peer mentor. The group size of 4–5 was chosen based on our own experience because very small groups sometimes fall apart, and doing research projects with larger groups makes it difficult to carve out meaningful, complimentary, individual project contributions as well as runs the risk of students feeling anonymous because it is more difficult for the students to get to know each other. Moreover, research supports this group size for capstone projects [2]. Mentors in our program are typically a year or two ahead of participants but belong to similar demographic groups and come from similar academic backgrounds. The mentor leads the team in learning skills and applying them to a research question. Each team also has a faculty advisor, who comes up with a research project that is likely to be completed in the available time and that is of interest to the students [3]. The faculty advisor meets with the whole team at least once per week to guide learning and research. Of note, acting as a mentor improves students’ retention and success in science, technology, engineering and math (STEM) [4], therefore this setup benefits mentors as well as mentees.

### 4. Find mentors and pay them well

Over the years, we have found that near-peer mentors are incredibly useful for a number of reasons, including that (1) student participants are more likely to ask for help from a near-peer mentor than from a faculty advisor, (2) near-peer mentors serve as role models, giving participants an idea of what they can aim for in the next year or two, and (3) the use of mentors allows the program to serve many more participants than it could if it relied on a few time-pressed faculty advisors. Our selection criteria for mentors include essential knowledge (for example, the mentor for a team doing an advanced chemistry research project should have taken physical chemistry), mentoring experience or potential, logistical availability, and having a similar demographic background as the participants. Mentors don’t need experience with the specific coding language or research topic they will work on with their team. Rather than being the expert in the room, they are expected to help team members work together to find solutions or formulate questions for the faculty advisor. The application for mentors is similar to that for program participants, but includes more information about research, programming, and mentoring experience. Thanks to the large number of mentor applications we have received each year, we have easily been able to select excellent mentors, most of whom are HU and/or women or gender nonconforming. Mentors are crucial for the success of the program and need to be paid well for their work. Each week of the program, we pay our mentors a competitive wage for 8 contact hours with their team, a 2-hour all-hands lunch meeting, a 2-hour mentor meeting, and 4 additional hours to account for preparation.

### 5. Identify an appropriate online course for each team

We have found that when learning basic programming skills, interactive online classes to learn computer programming (for example, from Datacamp [https://www.datacamp.com/], Udacity [https://www.udacity.com/], or Coursera [https://www.coursera.org/]) motivate and engage students better than books or online texts. Yet, when working individually, most students—especially beginners and HU students—don’t finish online classes [5,6]. As a solution, we have found that in teams, where students can work together and support each other, they learn a great deal from an online class. Each team’s faculty advisor picks a free, clearly structured online class to teach participants coding skills relevant to their research project. We have had good experiences with Udacity’s Exploratory Data Analysis course because this class is suitable for beginners. It does a good job motivating students to think about data and learn R. In early team meetings, participants spend time quietly working on the online class with their headphones on, followed by a team discussion or collaborative problem-solving session. If students encounter difficulty with any of the material, mentors may develop mini-lectures and create their own exercises to facilitate learning. Note that the students’ goal is not necessarily to finish the online course, but to learn enough to perform their research project.

### 6. Assign each team a simple and engaging research project

Learning to code without a specific application in mind can feel boring and irrelevant, sometimes leading students to abandon the effort. In our summer program, teams carry out a research project to motivate them to learn coding skills, improve their sense of belonging in science [7], and cultivate teamwork and time/project management skills. Faculty advisors assign each team a research project early in the program. These projects should answer real questions so that participants feel their work is valuable [8]. The projects should also be relatively simple. Small and self-contained projects that can be completed within a 3-week time frame are ideal to ensure completion and make participants feel that their efforts have been successful. For example, past research projects in our program, which reflect the interests of faculty advisors and the students, include writing computer simulations to model the evolution of gene expression, analyzing bee observations from a large citizen science project, examining trends in google search term data with respect to teen birth outcomes, and building an app for finding parking spots on or near campus.

## During the program

Both students and mentors need support, community, and a shared objective to thrive in the program.

### 7. Support the mentors

During the 8 hours per week that mentors spend with their teams, they have to troubleshoot a wide variety of problems, including technical issues, as well as personal and interpersonal issues such as reluctance to share ideas. We now train mentors before the program starts (in 2019, we offered a 2-day mentor training). In this training, mentors are equipped with tools for working with their team. For example, they learn to frequently gather anonymous feedback from their students (the mentor might prompt “write down one thing you learned and one thing that confuses you this week”). In addition, an experienced mentor advisor leads the mentors in a 2-hour weekly meeting to troubleshoot group dynamics and project hang-ups, discuss pedagogy, and respond to weekly student evaluations. These meetings are a crucial time for mentors to get feedback, hear from others running into similar issues, and share solutions. We have had the most success when an experienced advisor who is not a faculty advisor of any of the summer teams runs the mentor meetings, as it alleviates any pressure that some mentors may feel to hide their challenges. We also encourage mentors to communicate with their faculty advisor openly and regularly.

### 8. Run a weekly lunch meeting to facilitate a spirit of program-wide community

The first time we ran our summer program with multiple teams, we received overwhelming feedback that participants wanted to get to know students from other teams. Therefore, we instituted a weekly all-hands meeting during which all participants and mentors in the program get to meet and mingle. Importantly, we provide the food at these meetings and plenty of time for students to eat and chat. Sometimes we invite external speakers from nearby companies or universities to these meetings. We also use these meetings for the faculty involved in the program to talk about their research, and for a workshop on coping with stereotype threat that can lift the performance of women and minorities in STEM [9]. Our overall goal is to create an intellectually safe environment in which all social identities are welcome and affirmed as valuable in the practice of computational research.

### 9. Have teams present their research at the end of the program

During the last week of our summer program, each team creates and prints a scientific poster describing their research. They present these posters at a fairly large end-of-summer symposium where students from many summer programs on our campus share their work. This conference is a lively event with many students, faculty, and family members in attendance. Participants seem to develop a stronger identity as a scientist by preparing posters, seeing the professional printed products, and proudly presenting their work [10]. In fact, in our program-end survey, many participants report that the symposium is the highlight of the program, some citing the symposium as a transformative experience that spurs them to further pursue research. Thanks to a collective effort from faculty leading multiple summer research programs on our campus, these presentations were also designed to be an opportunity for participants to help demystify research for their family members who attend. The hope is that families who better understand young scientists’ accomplishments and ambitions are better advocates for their future research efforts. For institutions that lack the infrastructure for a large all-day symposium, a smaller program-based poster session could serve the same purpose.

## After the program

### 10. Help students identify their next step in CS

Many students are excited to do more coding, research, or both after the program ends. To connect students to future research opportunities, we dedicate the last all-hands lunch meeting to a networking session with principal investigators (PIs) on campus, many of whom are interested in meeting the summer students who now have 90 hours of coding and research experience. Each PI has 3 minutes to pitch a project. Then the faculty sit at tables, where students can approach them to talk about the projects. All interested alumni of the 2018 summer program joined research groups the following fall. In addition, we give students information about relevant courses at SFSU they can take that include some coding (e.g., an infectious disease class that involves coding in R, a forensic genetics computational lab class, or a Python introduction to coding class). SFSU also offers a 5-course CS minor for biology and chemistry minors, which is a great option for alumni from the summer program [11].

## Conclusion

Using these 10 simple rules, we have been able to set up a popular and effective program that uses few resources. The influx of students trained in our program has had a substantial impact on our primarily undergraduate-serving institution, allowing faculty and students to carry out many new research projects together. The program originally started in the Biology Department, but as our program has expanded, we have found it possible, though more complicated, to include projects that are of interest to students from economics, public health, and ethnic studies.

Our end-of-the-program survey, given to both participants and mentors, is used internally to improve the program year over year. We decided to write this 10-rule paper to share the ideas we have developed from our experiences running this program over a number of years. If you are planning to start a similar program on a low budget, know that you can start with just one team of students (4–5 undergrads). We hope you are inspired to adopt whichever of our rules make sense to create your own program customized to your specific institution. We would love to hear about your program as it develops!
